# Collectanea Medica: Consisting of Anecdotes, Facts, Extracts, Illustrations, &c.

**Published:** 1822-09

**Authors:** 


### COLLECTANEA MEDICA:

CONSISTING OF

### ANECDOTES, FACTS, EXTRACTS, ILLUSTRATIONS, &c.

*Relating to the History or the Art of Medicine, and the Collateral Sciences.*

---

Floriferis, ut apes, in saltibus omnia libant,

Omnia nos, itidem, depascimur aurea dicta.

---

ART. I.—*Cases illustrating the decided Efficacy of Cold Affusion in the Treatment of Poisoning from Opium.* By SEPTIMUS WRAY, Esq. Member of the Royal College of Surgeons, and of the Medical Societies of London, Montpellier, and Marseilles.

CASE 1.—I was called, early in January 1821, to Mrs. E——, who had, half an hour before, taken about two ounces of laudanum. I found her in bed, in a state of profound stupor. Her pulse was much quicker than natural; her pupils were dilated. Every means which could be suggested at the time were employed to rouse her from her lethargy, but without effect. Under such circumstances, no internal remedies could be administered. I afterwards had recourse to cold affusion, which produced the most decided benefit. A large bucketful of cold spring water was brought into the room, and a quart basinful was forcibly thrown on the head and chest. It roused her on the first application, but immediately afterwards she relapsed into the same state of stupor. By resorting repeatedly to the same means, in about ten minutes I had the satisfaction of hearing her speak. An emetic was then administered, which operated freely. Vinegar and water were given afterwards, and on the least tendency to drowsiness the cold affusion was repeated. I had the gratification, the following day, of seeing this lady perfectly restored.

CASE 2.—April 17, 1821. A gentleman, residing in the vicinity of Chancery-lane, took two ounces and a half of laudanum, in a fit of desperation, on account of some losses he had sustained. Immediately after taking it he became sensible of his folly, and informed the waiter of the coffee-house, where he was at the time, of the circumstance, who immediately sent for a medical gentleman. An emetic was instantly administered, and, after its slight operation, he was put into a hackney-coach, and driven to Fleet-street, where he had given his address. The coachman, on opening the door, found him lying at the bottom of the coach, in a state of perfect stupor, from which he could not be roused. He was taken in this state to the watch-house, where he was recognized, and thence conveyed to his own house; when another medical man and myself were sent for. That gentleman, having arrived some time before me, had employed the usual means in order to rouse him from the state of coma into which he had sunk. Every attempt produced merely a momentary effect; when left alone, he dropt into his former condition.

As soon as I arrived, I requested that the cold affusion to the head and chest might be tried. A few applications of it, in a similar manner as in the former case, had the effect of removing completely the profound stupor, and the other alarming symptoms which were present. He complained, the following day, of head-ach and soreness in the epigastric region: the former arising, most probably, from the effects of the opium on the nervous system; the latter from the irritation induced by the strong emetics administered in order to produce full vomiting. These symptoms soon yielded to bleeding and other antiphlogistic means.

CASE 3.—On the night of May 12, 1822, I was called to Mrs. W—, Whitefriars, an extremely delicate young woman, about twenty-five years of age, who, at half-past ten, had taken two ounces of laudanum, with the intention of destroying herself. Having been at that time particularly engaged, I sent my assistant, with directions to employ the cold affusion, and to administer an emetic as soon as deglutition might be accomplished. If the symptoms were very alarming, he was also instructed to send for me. Immediately after his arrival, I was again sent for, at his request. On entering the room, he acquainted me that he had considered it too late to do any thing, and therefore had not attempted it. She appeared, in fact, when I arrived, nearly dead. During the preparation for the cold affusion, I endeavoured to rouse her by various external means of irritation, but with no effect. The pupils were dilated, and quite insensible to the light from a candle that was presented close to them. The pulse could occasionally be felt in slight undulations, and the body possessed a considerable degree of warmth. The head and chest were raised, and I began by throwing a large basinful of cold water forcibly on the head, which produced an evident twitching in the muscles of the face. By repeating these means, at intervals of some seconds only, she uttered a lamentable scream, much resembling that of a person recovering from suspended animation by immersion. After a few more applications of the affusion, a very strong emetic was administered, with considerable difficulty; but it was no sooner taken than she relapsed into the same state of inanimation, from which she was only restored by the frequent and forcible dashing of the cold water on the head and chest. She was afterwards raised from the bed, and carried up and down the room between two persons, with nothing on but a chemise; and, by the repeated employment of the affusion, she might be said to have been in a continued shower-bath. In about half an hour from the exhibition of the emetic, it began to operate slightly. The ejected matters smelt strongly of laudanum. The vomiting was promoted by warm water and an additional emetic. After the stomach had been emptied, vinegar and water were freely administered. Notwithstanding these means had been used, she frequently relapsed into a state of syncope, from which she could only be roused by a fresh affusion. In about three hours from the commencement of the treatment, the pulse acquired greater force, and her appearance altogether showed an evident return of the powers of life. By constant attention, during six hours, to the means already employed, whenever they appeared requisite, I had the satisfaction to see her sufficiently restored to allow her, with perfect safety, a few hours of repose. She only suffered a little from debility, during two or three days.

*Remarks.*—The first time my attention was drawn to the use of the cold affusion in states of the system analogous to that which results from the immoderate ingestion of opium, was shortly before the occurrence of the case first related, when called to a patient in furious delirium, brought on by gin-drinking; and, as there was no possibility of taking away blood or administering internal remedies, I had recourse to the cold affusion, from a full conviction that the derangement was caused by a greater determination of arterial blood to the head, than could at once be carried to the veins, or returned by the lateral sinuses. Believing, also, that a continuance of this state will produce a distention of the capillary vessels in the brain, almost incompatible with life, owing to their delicate texture, and to the softness of the substance of the brain itself, I conceived the best plan to be employed was that which tends to prevent or overcome that distention.

From the well-known power that cold possesses of diminishing arterial action, and of producing constriction of vessels, I was led to its use in that case; and the effects which immediately followed its adoption exceeded my expectations.

The successful employment of the cold affusion in this case of extreme intoxication from a spirituous liquor, suggested its use in the treatment of poisoning from opium. The analogy between the one and the other I considered to be very close; the difference, in my opinion, consisting chiefly in the greater diffusibility of the latter agent, which would give rise to a more alarming degree of congestion of blood in the vessels of the head, after its primary effects had disappeared, than usually follows the ingurgitation of the former agent. The peculiar operation of opium upon the nervous system may also tend to produce a very different effect to that arising from the diffusible stimulants, when taken in an inordinate dose. This specific influence of opium, as well as its more common operation, I consider to be altogether destroyed by the shock made upon the system by the cold affusion; and the extreme vessels become, in consequence of its constringing effects, roused to a healthy and tonic state of action. The event of the three foregoing cases sufficiently proves the efficacy of the cold affusion, in removing the noxious effects of this powerful agent. I have contented myself with giving an outline of their more prominent features. They are the only cases which have come under my observation since the mode of practice was suggested to my mind, in the manner I have mentioned; and although they are few, still they are most important, from their uniformly successful issue. The last case more especially evinced the surprising effect of this remedial agent; for, to a superficial inspection, dissolution had apparently taken place, before any means of restoration was employed.—(*Medical Repository,* July 1822.)

**Art. II.**—*A Case of Poisoning by Opium, in which the cold Affusion was successfully employed; with Observations on the Medical Management of similar Occurrences.* By James Copland, M.D. &c.

August 26, 1821, I was called, about half-past eleven at night, to Mrs. B—, a young widow, aged about twenty-four years, who had taken, about twenty minutes before ten, an ounce and a half of laudanum. Mr. Carroll, a very respectable practitioner in my vicinity, who had been sent for some time previously to my arrival, and who had requested that I should be called, had succeeded, with difficulty, in administering half a drachm of the sulphate of zinc. This, however, produced no effect. Five grains of the sulphate of copper were afterwards exhibited, which, when assisted by irritation of the fauces, caused a slight vomiting. I arrived at this time, and found the following symptoms very prominently marked:—Her pupils were moderately dilated, and her pulse was 140, small, weak, and irregular. Her extremities, especially the hands and feet, were rather cold. She could with difficulty be roused, for a very short time, from a state of profound stupor; and, when roused, the constant sighing which was present, and the other symptoms of oppression about the præcordia, sufficiently marked the inability of the heart fully to perform its functions.

I directed cold water to be dashed copiously, and with considerable force, upon her head. The first affusion roused her immediately, and so effectually, that she expressed herself, in answer to my inquiries, to be "relieved from the load and numbness which she felt in the brain." While observing the effect of the affusion, I noticed that a return of sensibility was introduced by a very deep sigh; and, when the cold water fell upon the neck and chest, it was rendered somewhat convulsive. Soon after she was thus effectually roused, copious vomiting spontaneously supervened; undoubtedly in consequence of the sensibility of the system being awakened, by the affusion, to the irritation of the emetics formerly exhibited. This was promoted until all ingesta were supposed to have been removed. Her pulse was still weak and quick, and occasionally irregular, but rather fuller than formerly. Two teaspoonsful of the following formula were ordered to be taken in a cup of strong coffee, whenever the oppression at the præcordia and sighing were urgent.

℞ Spirit. Lavandulæ comp. ℥vj.

Liquoris Carbonatis Ammoniæ, ℥ss. M.

The forcible affusion of cold water on the head was enjoined as soon as stupor supervened, whilst the neck and chest were ordered to be wiped perfectly dry immediately afterwards, and the rest of the body to be kept dry and warm. She was also directed to be placed occasionally between two persons, and to be walked up and down the room. At one o'clock in the morning I left her, in perfect confidence of finding a still greater progress towards recovery at my next visit.

I visited her again at seven o'clock on the morning of the 27th, accompanied by Mr. Carroll. The affusion had been resorted to thrice since I left her, owing to the supervention of stupor. It had the immediate effect of rousing her, for a considerable time, from her lethargic feeling, and of relieving her from the load she felt in her head.

The powerful stimulant, prescribed at the former visit, had also been taken three or four times.

At this visit, the pulse was about 100, and somewhat oppressed; there was no head-ach; the feet and hands were rather cold; considerable oppression was complained of at the region of the heart, and congestion of this organ was indicated by sensations of fulness and tightness in that situation, accompanied by frequent sighing, and pain in the left shoulder and clavicle. Some pain was also felt in the stomach, which was a little increased by pressure; the result, most probably, of the irritation of the emetics. In order to relieve these symptoms, and more especially the accumulation of blood about the heart, a dose of the stimulating remedy already mentioned was ordered, and blood was directed to be taken from the arm, to the amount of eight ounces. This treatment was instituted with the intention of placing the moving power and the body to be moved, as nearly as possible, in due proportion. The detraction of blood produced slight syncope, which was soon removed by the recumbent posture. She afterwards felt much better, and complained merely of weakness and of a slight pain about the left shoulder, which, however, was greater before the bleeding. The following draught was then prescribed:

℞ Mist. Camphoræ, ℥x.

Magnes. Sulphatis, ʒjss.

——— Carbon. ʒss.

Spirit. Ammon. fœtid.

——— Lavand. comp. āā ℥ss.

Syrup. Aurantii, ʒj. M.

Fiat haustus ter die sumendus.

She was allowed four hours of sleep after this visit, and afterwards any article of diet for which she might evince an inclination.

I saw her again the following day (Tuesday the 28th,) with Mr. Carroll. The aperient draughts had operated moderately, and very comfortably. The pulse was much stronger, and fuller. There was no head-ach, nor pain in any situation. Indeed, with the exception of languor, she felt in her usual health.

Tonics, &c. were prescribed; and on the following day she was able to walk out.—(*Medical Repository*, July.)

---

Art. III.—*On the most efficacious Means of Remedying the Effects of Opium, when taken in Poisonous Doses.* By John Hanmer Sprague, Fellow of the Royal College of Surgeons, London; Honorary Member of the Medical and Anatomical Society of Bristol, &c.

The truly distressing catastrophe which lately occasioned the death of the late Bishop of Armagh, by tincture of opium, or laudanum, having been given in mistake for some other medicine, (in which unfortunate case, the united skill of fashionable physicians proved of no benefit to the patient,) has naturally excited much public concern, and induced me, at this time, to earnestly solicit the attention of my professional brethren to what I have found to be the most efficient means of remedying the dangerous effects of opium. This potent, but invaluable, medicine is frequently taken by design, or administered by mistake, as in the instance of his lordship above referred to;* and there is every reason to believe that many deaths are occasioned by a failure of the means commonly made use of as antidotes. It must be granted that, whenever we are called to a case where opium has been taken in a poisonous dose, we should, without any delay, make use of the most powerful measures to counteract its deleterious influence. The materia medica furnishes us with many efficacious remedies, which, if timely administered, will generally prove successful; but, to produce so desirable a result, they must be given in proper doses, and promptly and energetically applied. We are informed by authors, that "the first thing to be done is to endeavour to evacuate the contents of the stomach, by active emetics;" but, in such cases, that organ is rendered so insensible to stimuli, that all the emetics commonly advised to be given, such as sulphate of zinc, or copper, antimonial tartar, &c. frequently fail to produce vomiting, as must have been witnessed on many mournful occasions. The following is more to be depended on, and will seldom disappoint the anxious desire of the practitioner, in rousing the action of the stomach:

℞ Ammoniæ Subcarbonatis, ℈j.

Pulv. Rad. Ipecac. ʒss.

Aqua Menthæ Pip. ℥iij.

Tinctura Capsici, ʒij. M.

ft. Haustus emeticus, quamprimum adhibendus.

If the patient has lost the power of deglutition, the draught should be introduced into the stomach by means of a flexible hollow tube, or a large-sized gum-elastic catheter. Next, let a little of the liquor ammoniæ be taken on a feather, and put up the patient's nostrils; and a piece of folded linen, wetted with the same, laid over the region of the stomach, which sometimes raises an instantaneous blister, and always proves useful. A single drop of the liquor vol. cornu cervi (being less caustic than the liquor ammoniæ,) should be cautiously dropped into the external canthus of the eye, which, by being diffused over the globe, (by the action of the orbicularis palpebrarum muscle,) has the most beneficial effects. The patient's head should be kept in the most erect position, and folded cloths, dipped in the coldest water, constantly applied to it; whilst the lower extremities are to be immersed in water of as high a temperature as can be borne.† A sufficient time having elapsed after the emetic has been given, its operation should be promoted by giving a quart of warm water, in which has been mixed two teaspoonsful of flower of mustard. After the stomach has been com-

* Vide the *Observer* newspaper of Monday, May 13, 1822.

† Mr. Collier, of Norfolk-street, in the Strand, has lately communicated to the profession a mode which he proposes for effectually rousing the system in cases of poisoning by narcotics. His method consists in scattering some of the pubis dolichi prurientis over the body of the patient, particularly about the head, neck, and arms. The effect is said to be almost immediate.—*London Medical and Physical Journal,* for March 1822.

pletely emptied, a strong decoction of coffee should be taken frequently, prepared by boiling two ounces of coffee coarsely ground, and half an ounce of bruised mustard-seeds, in a pint and half of water, for three minutes, and strained: a teacupful should be given every half-hour, with a tablespoonful of lemon juice,* for the space of three or four successive hours. All the means before specified should be used as expeditiously as possible, and the following enema administered, as soon as it can be prepared:

℞ Ol. Tereb. Rect.
Ol. Ricini, āā ℥j.
Vitel. Ovi, q. s. Misce. Adde gradatim,
Decocti Avenæ, ℥x.
Sp. Ammoniæ Ar. ʒiij. Misce.
ft. Enema, quamprimum injiciendum.

If we are so fortunate as to rescue our patient from the impending death occasioned by the primary effects of the opium, we have afterwards to counteract the great degree of exhaustion which takes place as a secondary consequence, and also to obviate constipation of the bowels. The former is best effected by giving brandy, or other cordials, in small quantities, in strong coffee, at proper intervals, and by the use of the following medicine:

℞ Infus. Caryophill. ℥iss.

Sp. Ammoniæ Ar. ʒj.

Tincturæ Capsici, ʒss.

Syr. Zingib. ʒiss. Misce.

ft. Haustus, ter quaterve in die sumendus;—vel haustus sequentis.

℞ Infusi Anthemidis, ℥x.

Acidi Sulphurici Diluti, ♏ xx.

Tincturæ Aurantii,

——— Cardamomi Simplicis, āā ʒj. M. ft. Haustus.

The latter intention, namely, evacuating the bowels, and keeping them patent, is most effectually done by the subsequent formulæ:

℞ Fol. Sennæ,

Conservæ Menthæ Sativæ,† āā ℥ss.

Sem. Coriand. Contus. ℈ij.

Aquæ Ferventis, ℥viij.

Macera per horas duas, et cola.

℞ Infusi suprapræscrip. ℥vij.

Sodæ Sulphatis, ℥j.

Tincturæ Sennæ, ʒvj.

Sp. Ammoniæ Arom. ʒij. M.

ft. Mistura. Capiat partem 4tam secundis horis,—donec bene solutus sit alvus,—et pro re nata repetendam.

---

* As acids take up and increase the narcotic power of opium, they always augment the mischief produced by an overdose, if exhibited previously to the evacuation of the stomach.—See Review of Orfila on Toxicology, *London Medical Repository*, for January 1818.

† *Conserva Menthæ Sativæ.*

℞. Fol. Menthæ Viridis recentis, ℥j.

Sacchari purificati, ℥iij. Contund. probè simul, fiant Conserv.

In conclusion, to assist the convalescence, I would strongly recommend the shower-bath to be used every morning for a fortnight, which has an excellent effect in restoring the lost tone and energy of the system. I have thus endeavoured to concentrate the most decidedly efficacious practice in cases of poisoning by opium, and I can with the more confidence recommend the above treatment, as I have witnessed its complete success in several desperate cases, where the usual means resorted to had been tried, and entirely failed. Wishing this to be altogether a practical paper, I have carefully abstained from theorizing on the effects of opium on the human system, which, by interfering with my present intentions, would merely have had a tendency to have amused the speculative imagination. "Give me facts, said my Lord Judge; thy conclusions are but the guess-work of imagination, which puzzle the brain, and tend not to solve this mystery."—(*London Medical Repository*, August.)

